# Supplementary material for: Correlated anomalous phase diffusion of coupled phononic modes in a sideband-driven resonator
Source: Nat Commun. 2016 Aug 31;7:12694. doi: 10.1038/ncomms12694 (PMC5013651; doi:10.1038/ncomms12694)
Supplement: Supplementary Information — Supplementary Notes 1-2 [file ncomms12694-s1.pdf]

Supplementary Note 1. Renormalization of the parameters

The nonlinear part of the potential energy of the modes can be written as

$$U(q_1,q_2)=\frac{1}{2}\gamma q_1^2q_2^2+\frac{1}{2}\beta_{12}q_1^2q_2+\frac{1}{2}\beta_{21}q_1q_2^2+\frac{1}{3}\beta_{11}q_1^3+\frac{1}{3}\beta_{22}q_2^3+\frac{1}{4}\gamma_1q_1^4+\frac{1}{4}\gamma_2q_2^4 \qquad (1)$$

The first term on the right hand side of Supplementary Equation 1 represents dispersive coupling in which the energy depends on the product of the squares of the beam and plate displacements. A standard procedure of nonlinear dynamics shows that the time evolution of the slow complex vibration amplitudes $u_{1,2}$ is described by Eq. (2) in which

$$\gamma_{kl}=\frac{\gamma}{m_k}+\frac{\beta_{kl}^2}{m_k^2(\omega_l^2-4\omega_k^2)}+\frac{\beta_{lk}^2}{m_km_l(\omega_k^2-4\omega_l^2)}-\frac{\beta_{kl}\beta_{ll}}{m_km_l\omega_l^2}-\frac{\beta_{kk}\beta_{lk}}{m_k^2\omega_k^2}\quad (k\neq l) \qquad (2)$$

where $k,l$=1,2; there is no summation over repeated indices. Parameters $\gamma_{11}$ and $\gamma_{22}$ are also renormalized compared to the values $\gamma_1/m_1$ and $\gamma_2/m_2$, with the extra terms being also quadratic in $\beta_{kl}$; the corresponding standard renormalization can be found in textbooks on nonlinear dynamics. It is clear from Supplementary Equation 2 that the parameters $\gamma_{12}$ and $\gamma_{21}$ depend on the parameters of the cubic nonlinearity of the potential (Supplementary Equation 1) and the effective masses. The parameters of the internal mode nonlinearity that are directly measured in the experiment are $\gamma_{11}$ and $\gamma_{22}$, with the appropriate renormalization already incorporated.

We now discuss the driving term in equations (2) for the complex amplitudes $u_{1,2}$. Equations of motion (1) in the main text are written for the driving energy of the form of $-Fq_1q_2\cos\omega_F t$. In a more general case, the energy of the vibrational modes in the external field that is applied to the beam (mode 2) has the form

$$H_F = -(Fq_1\, q_2 + F'q_2)\cos\omega_F t \qquad (3)$$

Here, the second term comes from the linear polarizability of the beam, whereas the first term, as explained in the main text, comes from the nonlinear response with the energy in the field being bilinear in the coordinates of the modes.

With the driving-induced term in the Hamiltonian of the modes of the form (Supplementary Equation 3) and with account taken of the nonlinear mode potential (Supplementary Equation 1), the terms $F_1, F_2$ in Eqs. (2) for the complex mode amplitudes in the main text become

$$F_k = \frac{F}{m_k} - \frac{\beta_{21}}{m_k}\frac{F'}{m_2(\omega_2^2-\omega_F^2)} \qquad (4)$$

with $k = 1{,}2$. We note important relations

$$\gamma_{12}m_1 = \gamma_{21}m_2, \quad F_1m_1 = F_2m_2 \qquad (5)$$

As a consequence, $\gamma_{21}F_1 = \gamma_{12}F_2$. We used these relations when solving Eqs. (2). In particular, for $\Delta + \omega_c > 0$ we obtain for the scaled squared amplitude of the vibrations of mode 1

$$|u_1|^2 = \frac{\Gamma_2\omega_2F_1}{\Gamma_1F_2\tilde{\gamma}_{12} + \Gamma_2F_1\tilde{\gamma}_{21}}(\Delta + \omega_c) \qquad (6)$$

where $\tilde{\gamma}_{12} = \gamma_{12} + \frac{3\gamma_{22}\omega_1}{2\omega_2}$ and similarly $\tilde{\gamma}_{21} = \gamma_{21} + \frac{3\gamma_{11}\omega_2}{2\omega_1}$. The expression for $|u_2|^2$ is obtained from the expression for $|u_1|^2$ by interchanging the subscripts $1 \leftrightarrow 2$ and $12 \leftrightarrow 21$. The vibration frequency of mode 1 is $\omega_1 + \delta\omega$ with

$$\delta\omega = -\frac{\Gamma_1}{\Gamma_1 + \Gamma_2}\omega_c + \left[\gamma_{21} + \frac{3\gamma_{11}\Gamma_2F_1\omega_2}{2\Gamma_1F_2\omega_1}\right]\frac{|u_2|^2}{\omega_1}. \qquad (7)$$

The expressions for the squared vibration amplitudes and the vibration frequency can be put in the form that contains only parameters that are directly measured in the experiment. For example, Supplementary Equation 6 can be written as

$$|u_1|^2 = \frac{\Gamma_2\omega_2}{(\Gamma_1+\Gamma_2)\gamma_{21}}(\Delta+\omega_c)\left[1+(\Gamma_1+\Gamma_2)^{-1}\left(\frac{3\Gamma_1\omega_1\gamma_{22}}{2\omega_2\gamma_{12}}+\frac{3\Gamma_2\omega_2\gamma_{11}}{2\omega_1\gamma_{21}}\right)\right]^{-1} \quad (8)$$

and similarly for $|u_2|^2$. These expressions were used in Fig. 3.

From Eq. (2), we derive that the sum of the phase of the vibrations of the plate and the beam is uniquely determined by frequency and amplitude of the driving field,

$$e^{i(\phi_1+\phi_2)} = \sqrt{\frac{16\omega_1\omega_2\Gamma_1\Gamma_2}{F_1F_2}}\left(\sqrt{\frac{F_1F_2}{16\omega_1\omega_2\Gamma_1\Gamma_2}-1}-i\right) \quad (9)$$

Supplementary Note 2. Relation between frequency noise spectrum and anomalous diffusion

In the model described by Eq. (2), the eigenfrequencies of the two modes are assumed to be constant. However, as shown in Fig. 4d, the eigenfrequencies fluctuate. For incommensurate frequencies of the self-sustained vibrations and the modulation frequency $\omega_F$, the vibration phases are arbitrary and undergo diffusion while remain to be anti-correlated with each other. The fluctuations of the total phase of the two modes $\phi_1 + \phi_2$ do not accumulate in time. The accumulating part of the phase $\Delta\phi_2(t) = -\Delta\phi_1(t)$ is related to the noise $\xi(t)$ of the frequency $\omega_2$ by equation $\Delta\dot{\phi}_2 = \xi(t)/2$. The mean square deviation of the phase is expressed in terms of the noise power spectrum $\Xi(\omega) = \frac{1}{2\pi}\int_{-\infty}^{\infty} dte^{i\omega t}\langle\xi(t)\xi(0)\rangle$ as

$$\left\langle \Delta\phi_2{}^2(t)\right\rangle = \frac{1}{4}\int_0^t dt'\int_0^t dt''\int d\omega\,\Xi(\omega)e^{i\omega(t'-t''))} = \int_0^\infty \frac{d\omega}{\omega^2}\Xi(\omega)(1-\cos\omega t) \qquad (10)$$

For the frequency noise of the 1/f type

$$\Xi(\omega) = \xi_f\,\omega^{-\alpha}. \qquad (11)$$

From Supplementary Equation 10 we obtain Eq. (4) of the main text, which describes the anomalous diffusion of the phase variance for sufficiently long times. The coefficient $C_f$ in this equation is

$$C_f = \xi_f\int_0^\infty dz z^{-\alpha-2}(1-\cos z) \qquad (12)$$

We note that the anomalous diffusion occurs on times which are long compared to the reciprocal high-frequency cutoff of the power law (Supplementary Equation 11) and short compared to the reciprocal low-frequency cutoff of the power law (Supplementary Equation 11). This is in full agreement with our observations.
